# Gender differences in unintended anterior pelvic roll during primary THA in the lateral position

**DOI:** 10.1186/s13018-024-04811-y

**Published:** 2024-07-20

**Authors:** Andrew P. Kurmis

**Affiliations:** 1 Discipline of Medical Specialties, University of Adelaide, Adelaide, SA Australia; 2 Department of Orthopaedic Surgery, Lyell McEwin Hospital, Haydown Road, Elizabeth Vale, SA 5112 Australia; 3 College of Medicine and Public Health, Flinders University, Bedford Park, SA Australia

**Keywords:** Hip navigation, Technology assisted surgery, Pelvic tilt, Pelvic roll

## Abstract

**Background:**

Fundamental morphologic differences between male and female pelvises are historically recognised. Despite this, little consideration has been given as to whether or not conventional positioning supports used for primary total hip arthroplasties (THAs) performed in the lateral position do an equally effective job of maintaining the intended set up position when comparing genders. Given that recent research has highlighted that unintended pelvic roll occurs commonly during hip surgery, and that such movement may have a mechanically-deleterious consequence upon final construct performance and complication rates, this study was undertaken to explore the differences in pelvic roll between genders.

**Methods:**

The output of a high-precision, commercially-available, imageless intra-operative navigation system was prospectively-collected for 85 consecutive patients undergoing unilateral, primary THAs. These data were separated by gender and were utilised to determine differences in pelvic movement around a central sagittal axis.

**Results:**

Demographic data were similar between genders, with no between-group differences in mean BMI (*p* = 0.09) or indication for surgery (*p* = 0.66), however participating males (mean 68.04) were slightly younger than females (mean 73.31). The mean anterior pelvic roll for females was 9.50°, and for males 8.68°. There were no statistically significant independent correlations observed between gender (*p* = 0.21) and pelvic roll.

**Conclusion:**

The findings of this novel study do not suggest gender differences in the magnitude of unintended, intra-operative, anterior roll, even when corrected for BMI and surgical indication. Average roll of ~ 9° was demonstrated across both groups. An awareness of such positional change during THA surgery may reduce potentially-avoidable post-operative complications.

## Background

Even the earliest of human anatomists recognised morphologic differences in the fundamental shape and proportions of the male and female pelvises [1–5]. These structural differences impart several biologic and mechanical advantages [2, 3], some favouring one gender, and vice versa. Worldwide, the majority of total hip arthroplasties (THAs) continue to be performed in the lateral (or decubitus) position [5]. Accurate attainment—and maintenance—of the true lateral position is critical for definitive placement of the acetabular component [6, 7]. Unintended or unappreciated positional deviation from the true lateral position may have deleterious effects upon several post-operative metrics of THA function including bearing stability/dislocation, component wear, squeaking, range-of-movement and dislocation rate [7–10]. In all instances, the lateral orientation of the patient’s pelvis is held by some type of physical support, commonly in the form of anterior and posterior ‘bolsters’ [7, 8, 11]. At present, a true ‘gold standard’ positioning support does not exist and many variants are in common use [12]. No previous published research has confidently confirmed whether or not conventional pelvic positioning supports are appropriately used for both genders (and/or differing pelvic morphology) and provide balanced stability (i.e. maintenance of the desired lateral position) in each case. The purpose of this study therefore, was to explore differences in the ability of conventional ‘off-the-shelf’ pelvic positioning supports to maintain the initial set up position during performance of routine primary THAs between genders.

## Methods

Prospectively collected data from the local database of a single surgeon series’ of computer-navigated THAs were retrospectively interrogated. All registry data were patient de-identified at the time of index entry, thus no patient-identifying information were utilised or accessed for this study. A convenience sample of 85 consecutive computer-navigated primary THAs were extracted from the database which included adult patients (i.e. age at the time of surgery ≥ 18 years) undergoing unilateral procedures performed in the decubitus position at one of two metropolitan tertiary teaching hospitals. Patients undergoing excisional arthroplasty, revision procedures, procedures requiring customised implants, or simultaneous bilateral THAs were excluded, as were instances of local dysplasia. Local human research ethics committee requirements were met in the performance of this study.

The magnitude of pelvic roll—as previously defined as the angular change in position around a central sagittal axis [7]—was extracted manually for each patient from the available dataset. Collected data were tabulated separately into a Microsoft Excel (Microsoft Corporation; Redmond, WA, USA) spreadsheet and analyzed using the SPSS (IBM; Armonk, NY, USA) statistical software. Statistical significance was set a priori at 0.05. Chi-squared testing was employed to establish any differences due to indication for surgery (categorical data). Two-tailed, unpaired t testing was performed to establish differences between the two cohorts with regard to BMI, age at time of surgery, and magnitude of pelvic roll (continuous data).

### Surgical technique

**Fig. 1:**
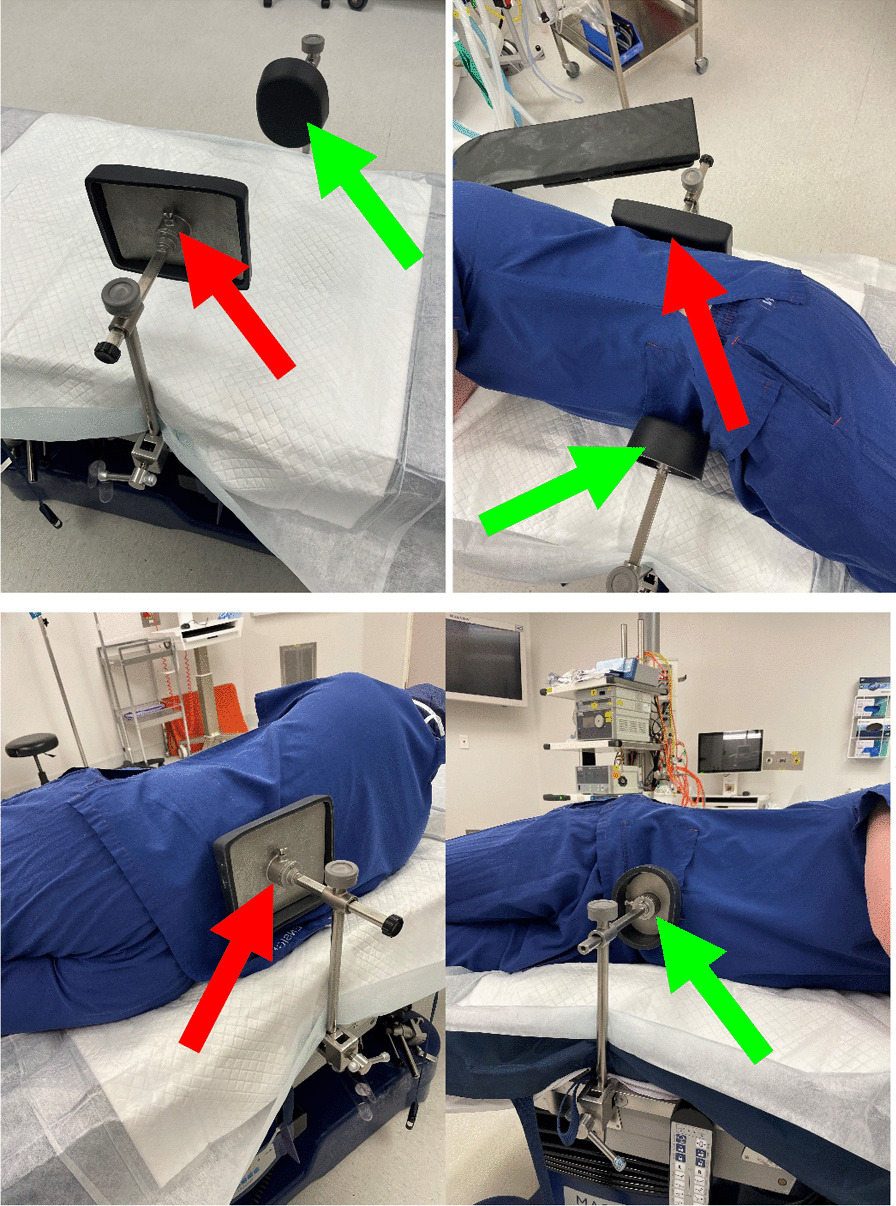
Positioning support set up demonstrating rectangular posterior (sacral) [red arrow] and rounded anterior (symphyseal) [green arrow] bolsters

After appropriate anaesthetic induction (GA or spinal), all THAs were performed in a lateral decubitus position. All operations (100%) were performed using routine positioning clamps which included a curved rectangular bolster overlying the sacrum posteriorly and a single-posted round anterior bolster positioned against the symphysis pubis (Fig. 1). The ‘true’ lateral starting position was determined using a composite of the verticality of the anterior superior iliac spines (ASISs) and sacrum, and a horizontal central gluteal cleft/fold. After pre-scrub, standard draping and skin prepping as per the local convention, the procedure commenced with insertion of a fixed pelvic tracker platform (Intellijoint; Intellijoint Surgical, Ontario, Canada) into the anterior element of the ipsilateral iliac crest using a previously published technique [8–13]. With the system camera applied to the proprietary tracker base, the pelvis was calibrated to the starting position [14]. The operative leg was returned to a ‘neutral’ position with the heels and knees superimposed to the underlying contralateral limb. This measure of orientation in three-dimensional (3D) space served as the ‘zero’ starting point and permitted later comparison for movement of the pelvis. It is noteworthy that attainment of a ‘true’ lateral decubitus position has no direct bearing on subsequent measurement, rather the difference between the initial and later positions was used to reflect unintended pelvic ‘movement’. The operation was then performed as per the clinical workflow of the operating surgeon.

The ‘final’ measurement of pelvic roll was performed at the end of the case—immediately prior to the initiation of closure—after the final implant construct had been inserted (and reduced) and the operative leg had been returned to a neutral position.

## Results

The results for all 85 cases were available for review. The cohort consisted of 48 females (56.5%) and 37 males (43.5%). There were no between group differences in mean BMI (*p* = 0.090) or indication for surgery (*p* = 0.660), although the male cohort (mean 68.04; SD 8.74) was slightly younger than the female cohort (mean 73.31; SD 9.12) at time of surgery (*p* = 0.004).

Across the entire cohort, the mean pelvic roll was 9.20° (range: 1.0–25.0°). Separated by gender, the mean anterior pelvic roll for females was 9.50° (SD 4.06), and for males 8.68° (SD 5.39). There were no statistically significant independent correlations observed between gender and the mean magnitude of unintended movement around the central sagittal axis (*p* = 0.212).

## Discussion

If unrecognised, unintended pelvic roll may lead to clinically-meaningful changes in final acetabular component version through perceptual distortion during terminal insertion. Such deviation from target position may compromise the mechanical characteristics of the final construct and has previously been associated with instability/dislocation, edge loading/bearing wear, squeaking, range-of-movement limitations and increased revision rate [7, 9, 15]. Especially while under sterile exclusion draping, the ability of surgeons or surgical teams to recognise such movement can be understandably challenging [7, 8].

For this hypothesis-generating investigation, we made a prospective decision to capture ‘final’ pelvic position data at the end of the surgical procedure—immediately prior to the commencement of soft tissue closure—such that the overall magnitude of patient roll could be determined. It was our two-fold intent to be able to demonstrate that clinically-meaningful unintended pelvic movement was indeed occurring during routine decubitus surgery and also to then quantify the overall magnitude of such movement in a uniaxial plane. We believe that we have been able to achieve both of these considerations in the current work with respect to gender differences (or rather lack thereof). We accept that pelvic roll throughout the case is unlikely to follow a linear progression, and that such movement is likely to be influenced substantially by force-imparting procedural steps such as primary retractor placement/leverage and acetabular reaming/cup impaction. We acknowledge that the amount of positional change likely has the greatest clinical relevance immediately prior to definitive acetabular component insertion—whereby such understanding might permit the surgeon to make corrective decisions regarding cup placement such that the intended final orientation appropriately considers/factors in pelvic roll from the starting position. Having demonstrated in our previous published work the common occurrence of anteriorly-biased pelvic roll during primary THA surgery in the lateral position [8], and having shown herein that patient gender does not appear to be a statistically-relevant confounder to the magnitude of such roll, we believe our presented evidence provides a scientifically-robust impetus to support future investigations aimed at more precisely describing the roll increments at sequential steps in the performance of a routine lateral THA. We hope to explore this element with targeted future studies.

**Fig. 2:**
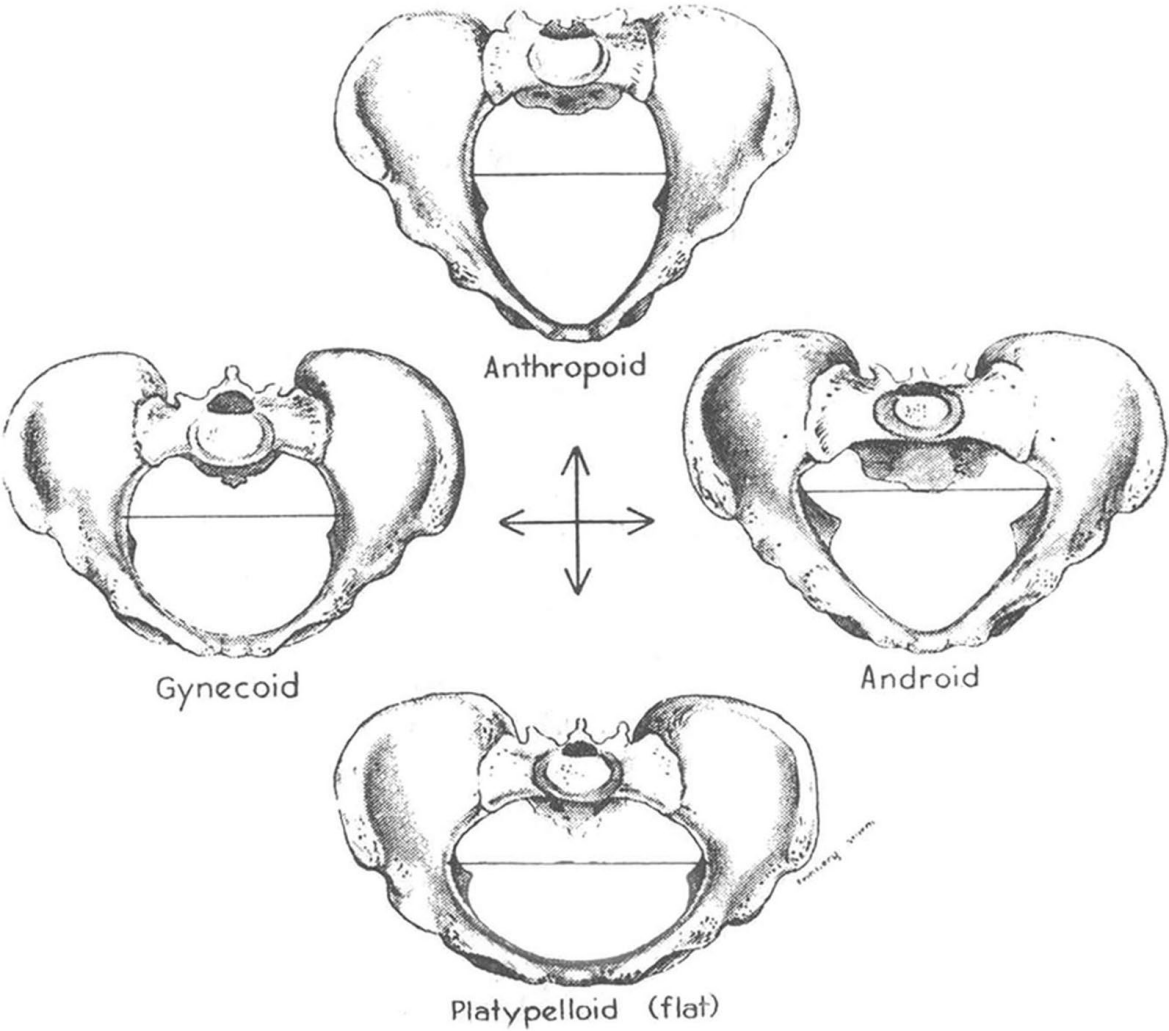
Recognised pelvic morpho-types, as described by Caldwell and Moloy [4]

Despite recognised differences in pelvic shape and proportions between genders [1], surprising little published work relates to such considerations being factored into pelvic position support design and/or application. There are four recognised pelvic ‘morpho-types’: gynecoid, android, anthropoid and platypelloid (Fig. 2) [4, 16]. The changing bony structure associated with these variants suggests that—when it comes to pelvic positioning clamps—that ‘one size may not fit all’. Despite this, none of the mainstream pelvic positioning supports marketed for THA use are offered as ‘gender specific’ nor with clear application differences for male and female patients. Despite the underpinning logic, in the absence of evidence of previous published work on the topic, we aimed to provide some initial data to explore whether or not there was a performance difference using standard positioning clamps during THA, as determined by the loss of angular position across the course of the procedure. We have previously shown—as have others—that unintended pelvic movement around a central sagittal axis (i.e. pelvic roll) commonly occurs during THAs performed in the lateral position [8, 12] and that such movement is almost universally anterior in vector [8]. We have also previously published work suggesting that BMI alone was not an independent correlate for the anticipated magnitude of pelvic roll [8]. While the ad hoc analysis of the relationship between the magnitude of intra-operative pelvic roll and patient BMI in the current study did not achieve statistical significance, we acknowledge that this consideration was not a primary evaluation goal of this work and the data pool itself was substantially underpowered to confidently demonstrate a true difference, if present. Future targeted studies of appropriate a priori size are indicated to further elucidate if a true difference between genders exists with regard to patient BMI at the time of surgery and the subsequent vector and magnitude of intra-operative pelvic movement in the lateral decubitus position.

This study has several potential limitations that require consideration. Firstly, despite the prospective collection of all of the data utilised in this study we cannot exclude a potentially biasing effect of the retrospective nature of the analyses we have performed. Secondly, while recognising that this investigation likely represents the first such reported initiative in the field (and hence cohort data do not exist to define ‘clinically meaningful difference’ ranges), it may well prove in time that the endeavour was under-powdered to unmask a true gender difference in pelvic roll that may otherwise reasonably exist—future work to validate our findings will add merit to this in due time. Thirdly, we have not considered potential ethnic nor racial variations in pelvic morphology and any possible influence this may have had on our results. While our recruitment was from a consecutive (i.e. non-selected) cohort of patients presenting to a high-volume arthroplasty centre, the potential for under-representation of particular racially-diverse groups cannot be excluded. Fourthly, we have deliberately limited our study to primary THAs performed by an experienced senior arthroplasty surgeon within a high-volume arthroplasty health network. The results we report here may therefore not be representative of those seen with more complex (and/or longer) procedures such as revision operations, when dealing with more challenging anatomy such the setting of dysplasia, or when performed by lower volume surgeons. Finally, while we elected to employ the standardised use of a commercially-available, off-the-shelf, set of paired anterior and posterior pelvic positional clamps for our study to maintain high internal validity we cannot claim generalisability necessarily to all other available brands/types used for the same purpose. A follow up investigation, using different supports may therefore yield different final results. We are happy with this reality for the current study as we are confident that any differences between genders (or lack thereof) represent patient variation, not confounded by support device differences. Based on the above, an opportunity does exist to externally validate our work in diverse patient populations and using different support devices.

## Conclusions

While unintended, intra-operative, anterior pelvic roll is a now recognised phenomenon, the findings of our novel investigation do not suggest that clinically-meaningful differences exist in such movement when comparing adult male and female patients. As with our previous work—and the independent work of others—our findings suggest an average anterior roll of approximately 9° during the performance of a primary THAs—again highlighting the surgeon’s need to be cognisant of this when performing routine hip operations, particularly at the point of definitive acetabular component insertion. Our study does not provide evidence to support suggestion of a need for ‘gender-specific’ positional supports, but may provide an impetus for future consideration of improved such devices that permit more stable pelvic positional hold.

## Data Availability

Not applicable.

## Abbreviations

3D: Three dimensional
ASISs: Anterior superior iliac spines
BMI: Body mass index
GA: General anaesthetic
SD: Standard deviation
THA: Total hip arthroplasty
